# Substantially increased sildenafil bioavailability after sublingual administration in children with congenital heart disease: two case reports

**DOI:** 10.1186/1752-1947-8-171

**Published:** 2014-05-30

**Authors:** Alexandra Carls, Julia Winter, Yeliz Enderle, Jürgen Burhenne, Matthias Gorenflo, Walter E Haefeli

**Affiliations:** 1 Department of Clinical Pharmacology and Pharmacoepidemiology, University of Heidelberg, Im Neuenheimer Feld 410, 69120 Heidelberg, Germany; 2 Department of Pediatric Cardiology and Congenital Heart Diseases, University of Heidelberg, Im Neuenheimer Feld 430, 69120 Heidelberg, Germany

**Keywords:** Enteral, Plasma concentration, Pulmonary hypertension, Sildenafil, Sublingual

## Abstract

**Introduction:**

Pulmonary hypertension is a progressive disease of diverse origin with devastating consequences in adults as well as in children. The phosphodiesterase 5 inhibitor sildenafil successfully lowers pulmonary vascular resistance. However, because of its poor enteral absorption, resulting in ineffective plasma concentrations, responses in infants and children are often erratic.

**Case presentations:**

We report the cases of two Caucasian boys, one born at term (case 1) and one aged 2.5 years (case 2), who had structural cardiac and pulmonary defects accompanied by symptomatic pulmonary hypertension. They received sildenafil enterally and sublingually and also intravenously in one of them. Plasma samples were taken at various time points to determine the plasma concentrations of sildenafil and its partially active metabolite. Sildenafil and *N*-desmethyl sildenafil were quantified using a validated liquid chromatography/mass spectrometry method. Oxygen partial pressure was determined from routine arterial blood gas samples.

**Conclusion:**

In agreement with previous observations in adults, we found that sublingual sildenafil was more extensively absorbed in our two pediatric patients. After sublingual administration, sildenafil plasma concentrations increased by 314% to 361% compared to enteral dosing. Concurrently, the metabolic ratio increased, suggesting not only that the overall absorption was enhanced but also that first-pass metabolism was partially bypassed. In case 2, the free fraction of sildenafil was 0.9%, which is considerably less than in adults (4%), suggesting that, in case 2, higher plasma concentration would have been needed to achieve effects similar to those in adults. Sublingual sildenafil appears to be a promising alternative route of administration in children with poor enteral absorption.

## Introduction

The phosphodiesterase 5 (PDE5) inhibitor sildenafil lowers pulmonary vascular resistance in patients with pulmonary hypertension (PH) [1]. Whereas enteral bioavailability in adults is about 40% [2], absorption in neonates and infants is often poor [3,4], thus limiting sildenafil’s effectiveness [5]. In pediatric cases, intravenous administration effectively increases exposure [6] and vasodilator responses [5]. However, intravenous administration is not practicable for long-term maintenance therapies.

In our present report, we describe the cases of two infants with poor enteral absorption of sildenafil. We observed that sublingual administration of sildenafil solution markedly increased exposure.

After obtaining written informed consent from the infants’ parents for compassionate use of sildenafil, we collected venous blood samples to evaluate sildenafil exposure (molar metabolic ratios (MRs) of sildenafil/*N*-desmethyl sildenafil, estimated area under the plasma concentration–time curve (AUC) and thus relative sublingual bioavailability (F)). Sildenafil steady-state concentrations (C_avg,ss_) were calculated by dividing AUC by the respective sampling period. Sildenafil, its partially active metabolite *N*-desmethyl-sildenafil and the free fraction (case 2) were quantified in plasma by using a validated liquid chromatography/mass spectrometry method (lower limit of quantification 1ng/ml) [7]. As a safety marker, we evaluated partial arterial oxygen pressure (pO_2_). Statistical and pharmacokinetic analyses were carried out using Prism statistical software version 6.0 (GraphPad Software, San Diego, CA, USA).

## Case presentation 1

A Caucasian boy born at term (birth weight = 3610g) became cyanotic 1 hour after birth. Despite continuous positive airway pressure (CPAP) support, his oxygen saturation continued to decrease, requiring the use of mechanical ventilation. Echocardiography revealed a persistent foramen ovale and an open ductus arteriosus Botalli with systolic right-to-left shunting. An open lung biopsy confirmed the suspected diagnosis of alveolar capillary dysplasia.

Because of the infant’s worsening pO_2_ level, sildenafil treatment was started 6 weeks after birth with an enteral dose of 2mg four times daily via a nasogastric tube (using an in-house preparation according to a method described previously [8]), which was increased to 4mg four times daily 2 days later. Four weeks later, weaning from mechanical ventilation was initiated, supported by intermittent CPAP and nasal oxygen (FiO_2_ = 35% to 60%, flow rate = 3l/min to 41/min). The patient’s pO_2_ level was highly variable, ranging between 46mmHg and 102mmHg (mean ± SD = 70.9mmHg ± 17.5mmHg), suggesting that the sildenafil exposure was either variable or ineffective. Concurrently, the boy was also maintained on ambrisentan, midazolam, chloral hydrate, clonidine, teicoplanin, ceftazidime, levetiracetam, nystatin, ethacrynic acid, furosemide, spironolactone, metolazone, acetaminophen, levomethadone, colecalciferol, sodium fluoride, levothyroxine, acetylcysteine, salbutamol and omeprazole. He also received phenobarbital, a strong cytochrome P450 enzyme inducer that could possibly lower sildenafil exposure.

**Figure 1:**
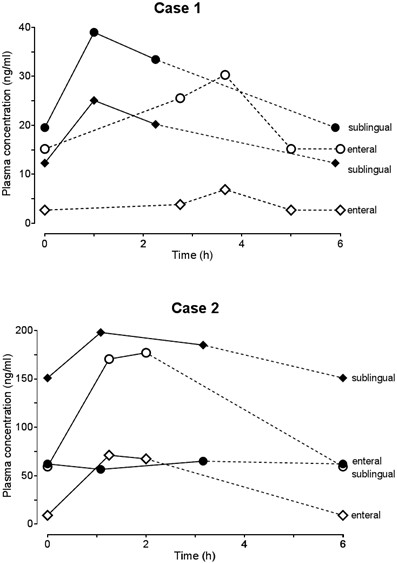
**Graphs depicting plasma concentrations of sildenafil and *N *-desmethyl sildenafil.** Sildenafil (open diamonds) and its major, partially active metabolite *N*-desmethyl sildenafil (circles) after enteral (open symbols) and sublingual administration (closed symbols) to two infants. The dotted lines are extrapolated assuming that plasma concentrations during steady state are identical at different dosing intervals.

Plasma samples taken during three different dosing intervals of 2.75, 3.6 and 5 hours after dosing showed very low concentrations (Figure 1). Assuming that plasma concentrations were obtained during steady state, we combined these samples to estimate the AUC as a marker of drug exposure (Table 1).

**Table 1:** **Pharmacokinetic parameters of sildenafil and *N *-desmethyl sildenafil**^**a**^

**Case**	**Sildenafil AUC**_**0–6h**_**((ng/ml)×h)**			***N*-desmethyl sildenafil AUC**_**0–6h**_**((ng/ml)×h)**			**MR**			**Absolute bioavailability (%)**		
	**IV**	**Ent**	**SL**	**IV**	**Ent**	**SL**	**IV**	**Ent**	**SL**	**IV**	**Ent**	**SL**
1	–	5.76	26.6	–	31.8	42.8	–	0.18	0.6	–	–	–
2	919	25.5	106	70.4	74.7	37.2	12.7	0.33	2.78	100	2.78	11.6

^a^AUC_0–6h_, Estimated area under the concentration–time curve in a 6-hour dosing interval, normalized to dose; IV, Intravenous; Enteral; SL, Sublingual; MR, Metabolic sildenafil/*N*-desmethyl sildenafil ratio.

We then switched sildenafil to sublingual doses administered with a small syringe (4mg/2ml four times daily). Sildenafil plasma concentrations in blood samples taken after 4 weeks on this regimen were substantially higher (Figure 1) than those after enteral administration. C_avg,ss_ increased by 361% and C_avg,ss_ of *N*-desmethyl sildenafil increased as well, albeit by only 35%, resulting in an approximately threefold higher MR (Table 1). During sublingual sildenafil, mean pO_2_ was 85.9mmHg (±27.2). By then, the patient was weaned from CPAP and received nasal oxygen (FiO_2_ = 100%, flow = 0.5l/min to 11/min). Nine days after being switched to sublingual sildenafil treatment, the boy was discharged from the hospital in an improved and stable respiratory condition, with sildenafil continued at home. No adverse drug reaction occurred during sildenafil administration.

## Case presentation 2

Case 2 is that of a 2.5-year-old boy (body weight 11kg) who had been treated with a hybrid approach (stenting of the ductus arteriosus and bilateral pulmonary artery banding) at the age of 6 weeks. Upon presentation to our hospital, he was diagnosed with atrial and ventricular septum defects, hypoplasia of the left ventricle, aortic isthmus stenosis, mitral valve insufficiency and persistent left superior vena cava. He underwent a Glenn procedure combined with Damus-Kaye-Stansel anastomosis to enlarge the systemic outflow tract, which resulted in a functional univentricular heart. Owing to low pO_2_, an additional 3.5mm modified Blalock-Taussig shunt was built. After he was weaned from cardiopulmonary bypass, he developed severe hypoxia, was unresponsive to hyperventilation and inhaled nitric oxide. Therefore, continuous intravenous sildenafil was started (0.088mg/kg/h, approximately 23mg/d) and delivery was switched to sublingual administration (8mg/4ml four times daily), which was increased to 10mg/5ml four times daily after 4 days. His clinical condition improved, and, 3 weeks later, sublingual administration was switched to enteral administration (10mg four times daily) via a nasogastric tube to facilitate administration of the large volume of the suspension. Concurrently, the child was also maintained on milrinone, dobutamine, metildigoxin, epinephrine, clonidine, neostigmine, furosemide, lisinopril, acetaminophen, levomethadone, simethicone, omeprazole, caspofungin and ceftazidime. He also received erythromycin and fluconazole, two strong cytochrome P450 enzyme inhibitors that can increase sildenafil concentrations, and metamizole, an enzyme-inducing drug that can potentially lower sildenafil exposure.Three blood samples were taken during intravenous infusion and after sublingual and enteral administration (Figure 1). Routinely drawn arterial blood gas samples were collected, and the oxygenation values obtained on 3 consecutive days were averaged as a composite marker for safety.

The highest sildenafil plasma concentrations were achieved during intravenous administration (Figure 1 and Table 1). After sublingual administration, C_avg,ss_ increased by 314% compared to enteral administration. Concurrently, the AUC of *N*-desmethyl sildenafil decreased by 50%, leading to an eightfold increase of the MR (Table 1). Compared to the bioavailability after intravenous administration, enterally delivered sildenafil bioavailability was 2.8% and four times higher than after sublingual administration (Table 1). The unbound fraction of sildenafil was 0.9%.

After extubation on the ninth postoperative day, oxygen was supplied via a high-flow nasal cannula (flow = 8l/min to 12l/min, which could be reduced over time to 41/min), which led to stable pO_2_ during sublingual (38.8 ± 4.05mmHg) and enteral (38.7 ± 8.61mmHg) sildenafil administration. Sildenafil was well-tolerated, and the patient was discharged from the hospital 28 days later, with enteral sildenafil continued at 10mg four times daily.

## Discussion

In pediatric patients, congenital heart diseases, when combined with pulmonary vascular diseases, are frequently accompanied by a large circulating blood volume, flooding the lungs through shunts and causing severe PH [1,6]. In these children, intravenous sildenafil reduces pulmonary arterial pressure and shortens the time to extubation and the length of postoperative intensive care [6]. Barst and co-workers have recently shown dose-dependent hemodynamic effects of oral sildenafil in children with PH (*n* = 235, ages 1 to 17 years). Because there was no change in mortality over a 16-week time period in their open-label extension study, their results revealed that children receiving the highest doses of sildenafil over 3 to 7 years had a higher mortality rate than those receiving medium or low doses of sildenafil (14% and 9% versus 20%, respectively) [9]. As a consequence, the US Food and Drug Administration (FDA) issued a warning label against the use of sildenafil in children [10]. As subsequently pointed out by Abman and co-workers, however, withholding lower doses from children may not be appropriate. Because the observed risks that led to the FDA’s issuing a warning label occurred only after several years of treatment, we did not consider the FDA warning label relevant for short-term use in patients such as ours [11]. When administered enterally, however, absorption is erratic. In almost half of these patients, sildenafil exposure is low and likely ineffective [3-5].

In adults with erectile dysfunction, sublingual sildenafil was at least equally effective as enteral sildenafil, and the onset of action after sublingual administration was more than 30 minutes faster than after enteral delivery [12]. In patients with end-stage heart failure, sublingual sildenafil decreases pulmonary pressure by approximately 20% [13]. Altogether, these results confirm rapid mucosal absorption. Sublingual pharmacokinetics have been assessed only in healthy volunteers, in whom bioavailability was found to be increased by roughly 50% compared to enteral administration [14].

In both children described in our present report, sublingual administration substantially increased sildenafil exposure. In case 2, absolute bioavailability could be estimated, and, though still low after sublingual administration (about 12%), it was four times greater than after enteral administration. Compared to adults (bioavailability = 36% to 47%) [2], enteral bioavailability in our pediatric patients was considerably lower, indicating that additional causes of impaired absorption may have been present. In adults, sildenafil absorption depends only minimally on gut content and food [2]; therefore, enteral feeding of our patients was not likely a cause of the limited enteral bioavailability observed.

After sublingual administration, the MR increased, suggesting not only that net absorption was enhanced but also that first-pass metabolism was partially bypassed. In healthy adults, the MR has been reported to be between 1.8 and 2.6 after enteral administration [2,15,16]. In the cases of comparable children described in previous publications, the MRs were 1.1 and 1.6 after enteral administration [3,4] (that is, substantially larger than in our cases). These findings suggest extensive enteral or hepatic first-pass metabolism, which is unexpected in infants, who generally do not yet have fully developed oxidative metabolic capacity of the relevant cytochrome P450 isozyme CYP3A4 [17]. However, both children in our present report were maintained on enzyme-inducing agents (phenobarbital or metamizole [18]), probably partly explaining the differences in MR observed between sublingual and enteral administration. Because *N*-desmethyl sildenafil is a 2.5-fold weaker PDE5 inhibitor than the parent compound [19], the net vasodilating effect is expected to increase with sublingual administration.

Sildenafil pharmacokinetics in severely ill neonates and infants are variable, and optimal target plasma concentrations are unknown. Indirect evidence suggests that total plasma concentrations greater than 100ng/ml in adults are needed for effectiveness [20]. Sildenafil is highly bound to albumin and α_1_-acid glycoprotein (96%) in adults (4% free fraction), and binding depends on age. In young males, the unbound fraction was reported to be 26% higher than in elderly individuals [16]. In our second case, the free fraction was high enough to be quantified and was surprisingly low (0.9%). If the concentration–response curve is similar across all age ranges, then it appears possible that, to achieve optimal effects, infants will require higher total plasma concentrations than adults.

Besides the fact that information in case reports is limited in general, another limitation is worth mentioning. We collected few blood samples, and the estimated enteral AUC value in the first case was composed of samples collected in three consecutive dosing intervals. Although this strategy confirmed that the exposure with sildenafil was consistently low, it assumed that pharmacokinetic variability between doses is small, which is unknown in this population. Finally, our findings suggest that sublingual sildenafil may become a promising route of administration in pediatric patients. However, its use cannot be broadly recommended before thorough pharmacokinetic data are available. In addition, a formulation with a higher sildenafil concentration probably also should be developed, because the sublingual volumes may become large if high sildenafil doses are to be administered.

## Conclusion

Switching from enteral to sublingual administration in two infants with poor enteral absorption of the drug substantially increased sildenafil exposure and favorably shifted the MR toward higher concentrations of the more active parent compound. These findings suggest that the sublingual route of administration may be a promising way to increase sildenafil exposure in the many pediatric patients with poor enteral availability.

## Consent

Written informed consent was obtained from both patients’ parents for publication of this case report and any accompanying images. A copy of the written consent is available for review by the Editor-in-Chief of this journal.

## Abbreviations

AUC: Plasma concentration time curve; C_avg,ss_: Steady-state concentration; CPAP: Continuous positive airway pressure; FiO_2_: Fraction of inspired oxygen; FDA: US Food and Drug Administration; MR: Molar metabolic ratio; PH: Pulmonary hypertension; PDE5: Phosphodiesterase 5; pO_2_: Partial arterial oxygen pressure.

## Competing interests

The authors declare that they have no competing interests.

## Authors’ contributions

AC, WEH and MG made substantial contributions to the conception of sublingual administration of the drug to achieve higher systemic availability. AC, JW, MG and WEH acquired, calculated and interpreted the data and related pharmacology to clinical outcome. YE and JB were responsible for the biochemical analysis of the blood samples. AC and WEH were the major contributors to the writing of the manuscript. JW, YE, JB and MG were involved in drafting parts of the manuscript and revising it critically for clinical and pharmacological content. All authors read and approved the final manuscript.
